# Investigating Temporal Patterns of a Native Bee Community in a Remnant North American Bunchgrass Prairie using Blue Vane Traps

**DOI:** 10.1673/031.012.10801

**Published:** 2012-09-06

**Authors:** Chiho Kimoto, Sandra J. DeBano, Robbin W. Thorp, Sujaya Rao, William P. Stephen

**Affiliations:** ^1^ Oregon State University, Hermiston Agricultural Research and Extension Center, 2121 S. 1^st^ Street, Hermiston, OR 97838; ^2^ Department of Fisheries and Wildlife, Hermiston Agricultural Research and Extension Center, Oregon State University, Hermiston, OR 97838; ^3^ Department of Entomology, University of California, Davis, CA 95616; ^4^ Department of Crop and Soil Science, Oregon State University, Corvallis, OR 97331

**Keywords:** bee monitoring, community composition, grasslands, native bees, Pacific Northwest Bunchgrass Prairie, temporal variability

## Abstract

Native bees are important ecologically and economically because their role as pollinators fulfills a vital ecosystem service. Pollinators are declining due to various factors, including habitat degradation and destruction. Grasslands, an important habitat for native bees, are particularly vulnerable. One highly imperiled and understudied grassland type in the United States is the Pacific Northwest Bunchgrass Prairie. No studies have examined native bee communities in this prairie type. To fill this gap, the bee fauna of the Zumwalt Prairie, a large, relatively intact remnant of the Pacific Northwest Bunchgrass Prairie, was examined. Native bees were sampled during the summers of 2007 and 2008 in sixteen 40-ha study pastures on a plateau in northeastern Oregon, using a sampling method not previously used in grassland studies—blue vane traps. This grassland habitat contained an abundant and diverse community of native bees that experienced marked seasonal and inter-annual variation, which appears to be related to weather and plant phenology. Temporal variability evident over the entire study area was also reflected at the individual trap level, indicating a consistent response across the spatial scale of the study. These results demonstrate that temporal variability in bee communities can have important implications for long-term monitoring protocols. In addition, the blue vane trap method appears to be well-suited for studies of native bees in large expanses of grasslands or other open habitats, and may be a useful tool for monitoring native bee communities in these systems.

## Introduction

Pollination is one of the most important ecosystem functions that animals fulfill. It is estimated that over 75% of the 250,000 species of flowering plants, including crops that make up 35% of the world's food supply, are pollinated by animals (Klein et al. 2007; NRC 2007). One of the most important groups of pollinators is bees. Bees pollinate many different crops and wild plants efficiently (Batra 1995; Klein et al. 2007). Their pollination of agricultural crops is of great economic benefit, with an estimated value of billions of dollars annually (Losey and Vaughan 2006; NRC 2007). In addition to their economic value, the fitness of many cross-pollinated, non-cultivated plants depends on bee pollination. Even plants capable of self-pollination may benefit from pollinators through higher seed set and a reduction in inbreeding depression (Michener 2007). Thus, bee pollinators are essential to maintaining the genetic diversity of many plant species.

There is evidence suggesting that we are currently in the midst of a global pollinator crisis, in which many invertebrate pollinator species are experiencing large declines (Buchmann and Nabhan 1996; Kearns et al. 1998; Säo Paulo Declaration on Pollinators 1999; NRC 2007). These declines not only include the domestic honey bee (*Apis mellifera*), but also native bees that act as important pollinators in both natural and agricultural systems (Allen-Wardell et al. 1998; Kearns et al. 1998; Biesmeijer et al. 2006; NRC 2007). Although less well-studied than domestic honey bees, there are several reasons why some native bees are declining. First, while native bees may not be sensitive to many of the same parasites and pathogens that are currently impacting *A. mellifera*, they are impacted by others, such as a fungus, *Nosema bombi*, and a protozoan, *Crithidia bombi*, that infect bumble bees (Thorp 2003; Rao and Stephen 2007). Other factors that may be negatively impacting native bees are the overuse of insecticides, and the introduction of non-native species, such as *A. mellifera* (Kearns et al. 1998; Thomson 2004; NRC 2007). Finally, native bees can be negatively impacted by habitat destruction and degradation caused by human activities, such as urban development, construction of roadways, and agriculture, including crop production and livestock grazing (Michener 2007; NRC 2007). The impact of crop production and grazing is particularly pervasive, especially in grassland habitats. Historically, 42% of the land surface on earth was covered with grasslands; this coverage is now less than 13% (Smith and Smith 2000). Thus, documenting the diversity of pollinator communities in these threatened habitats and monitoring them effectively for future changes are pressing conservation priorities.

One of the most threatened and understudied grasslands in North America is the Pacific Northwest Bunchgrass Prairie, which historically covered over eight million hectares in Oregon, Washington, Idaho, and Montana in the U.S.A., and British Columbia and Alberta in Canada (Tisdale 1982). Over 90% of this unique grassland type has been converted to agriculture, yet little is known about the native bee communities that inhabits it. Although several studies have examined native bee communities and their temporal variability in tallgrass and shortgrass prairies in the Great Plains region of the U.S.A. (Tepedino and Stanton 1981; Hines and Hendrix 2005; Davis et al. 2008; Kwaiser and Hendrix 2008; Kearns and Oliveras 2009), and general descriptions of bee fauna of the Pacific Northwest and California exist (Stephen et al. 1969; Moldenke and Neff 1974; Thorp et al. 1983), no published work has described native bee communities of the Pacific Northwest Bunchgrass Prairie. Here, the results are presented from a study examining the native bee community in this grassland type, at the Zumwalt Prairie in northeastern Oregon.

Because of its moderately high elevation (> 1500 m), short growing season (< 150 days), and aridity (precipitation < 50 cm/year), the Zumwalt Prairie has largely escaped conversion to cropland, and thus is one of the largest (∼ 65,000 ha) remaining remnants of the Pacific Northwest Bunchgrass Prairie (Kennedy et al. 2009). In addition, the Zumwalt Prairie also contains the largest known population of a threatened plant species, *Silene spaldingii*, which requires pollinators to maintain viable populations (Lesica 1993; Taylor et al. 2009). Effectively managing this species, as well as Pacific Northwest Bunchgrass Prairie in general, depends on acquiring baseline data on the abundance and temporal variability of native pollinators.

This study also used a relatively new trapping technique–blue vane traps–which has not been used in grassland habitat before. Although originally designed for beetle collection, Stephen and Rao (2005, 2007) discovered that these traps also attract a variety of native bees and have used them in several studies of agroecosystems (Stephen and Rao 2005, 2007; Rao and Stephen 2009, 2010; Stephen et al. 2009). This trapping method adds another option for sampling native bees to the traditional methods used that include sweep- and hand-netting, visual observations in plots or along transects, pan traps, and trap nests (reviewed by Westphal et al. 2008). Evidence collected in agricultural systems suggests that blue vane traps may be more efficient at collecting greater numbers of individuals and species than some techniques, such as netting, and that the traps have some logistical advantages as well (Stephen and Rao 2007).

The objectives of this study were to use blue vane traps to (1) describe the taxonomic composition, richness, diversity, and sex ratios of the bee community of the Zumwalt Prairie of northeastern Oregon, and (2) quantify seasonal and inter-annual variation in these characteristics for the overall grassland community as well as at the trap level.

## Materials and Methods

### Study area

The study was conducted within The Nature Conservancy's (TNC) 13,269 ha Zumwalt Prairie Preserve (45° 34′ N, 116° 58′ W) in Wallowa County of northeastern Oregon, U.S.A. Although the Zumwalt Prairie has been used as summer pasture for horse, sheep, and cattle for over 100 years, the majority of the area remains dominated by native species with most ecological processes still intact, and is considered to be an important refuge for an array of native biodiversity, including vertebrates, invertebrates, and plants (Kennedy et al. 2009). The Zumwalt Prairie is dominated by native grass species including Idaho fescue (*Festuca idahoensis*), Sandberg bluegrass (*Poa secunda*), prairie Junegrass (*Koeleria macrantha*), and bluebunch wheatgrass (*Pseudoroegneria spicata*) (Kennedy et al. 2009). In addition, over 112 forb species have been documented in the Zumwalt Prairie Preserve (http://conserveonline.org/workspaces/ZumwaltPrairieWorkspace/documents/zumwalt-prairie-plant-list-.pdf/view.html).

**Figure 1.**
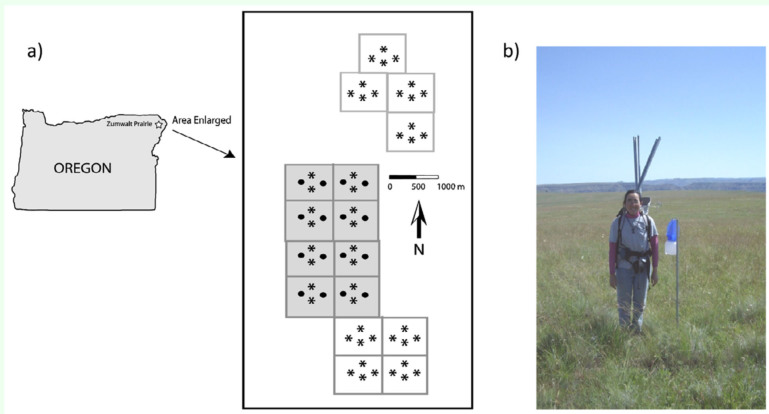
(a) Location of the Zumwalt Prairie in northeastern Oregon and location of pollinator traps in each pasture (sites denoted with “•” and shaded pastures were sampled in each season of each year, and traps denoted with “*” and unshaded pastures were not sampled in June 2007) and (b) a blue vane trap. High quality figures are available online.

The study was conducted in sixteen 40-ha study pastures on a plateau located in the center of the TNC Zumwalt Prairie Preserve (Figure 1a). The study pastures are part of a long-term study aimed at understanding the impact of livestock grazing on grassland food webs (Johnson et al. 2011). Pastures were selected based on relative uniformity of vegetation and topography, and were exposed to differing levels of cattle grazing (see Kimoto et al. in press, for details), resulting in a matrix of varying livestock utilization in the study area that is typical of the current land use in the larger Zumwalt Prairie (Bartuszevige et al. 2012).

### Bee sampling

Bees were sampled during the summers of 2007 and 2008 in the sixteen 40-ha study pastures with ultra-violet reflective blue vane traps (Figure 1a, 1b). Blue vane traps consist of a plastic container (15 cm diameter × 15 cm high) with a blue polypropylene screw funnel with two 24 × 13 cm semitransparent blue polypropylene cross vanes of 3 mm thickness (SpringStar™ LLC, www.springstar.net) (Stephen and Rao 2005). Traps were suspended approximately 1.2 m from the ground with wire hangers inserted into aluminum pipes (Figure 1b). No liquids or other killing agents were used in traps.

Bees were sampled during two bouts in 2007 (18–21 June and 9–21 July) and three bouts in 2008 (7–16 June, 10–18 July, and 25–29 August). In June 2007 bees were sampled using 16 blue vane traps in eight pastures; for all other sampling bouts, 64 traps in 16 pastures were used (Figure 1a). Elevation of traps ranged from 1372 to 1499 m. In 2007, traps were left open for two consecutive days and, because of high efficiency demonstrated in the first year, in 2008 they were left open for one day. Bees collected in the traps were frozen until they could be pinned, labeled, sexed, and identified to species, if possible, or morphospecies, if species identification was not possible. *Bombus californicus* and *B. fervidus* were treated as two separate species, although uncertainty over their status is ongoing (Williams 2010). Representative specimens of all species and morphospecies were vouchered at the Oregon State Arthropod Collection at Oregon State University in Corvallis.

### Floral resources and weather

Data on the presence and abundance of blooming forbs and weather were also collected. Data were collected on the presence and abundance of blooming forb species in 2008 along 50 m long, 0.3 m wide belt transects centered on each blue vane trap. The species and number of stems of each blooming forb that fell within the belt transect were recorded during each sampling period. Weather data were collected at a weather station located in the center of the Zumwalt Prairie Preserve (45° 34′ 39.88″ N, 116° 58′ 18.31″ W, elevation 1337 m) and less than 3 km from the nearest blue vane trap.

### Data summary and analyses

To describe the overall native bee community of the Zumwalt Prairie Preserve grassland and how it changes through time, the community was characterized with respect to abundance, richness, evenness, diversity, and species composition. Because sampling effort varied, both with regard to the number of traps used for each sampling bout and the time they operated, bee abundances were standardized by expressing them as the number of bees collected per trap per hour of daylight (henceforth referred to as “adjusted abundances”). Taxon richness, evenness, and Shannon diversity were calculated for each season in each year. In addition, in order to compare taxa richness among samples that varied in abundance, species richness estimates were generated by calculating the Chao1 richness estimator with log-linear 95% confidence intervals (Chao 1987). When examining temporal patterns of the entire native bee community of the Zumwalt Prairie Preserve, the sample size was one, so no statistical analyses were conducted.

To examine temporal variability in bee abundance and adjusted richness at the trap level, one-way ANOVA were conducted with time as the factor, using each trap as a replicate. The purpose of this analysis was to determine if spatial variability at the scale of the trap (represented in the error term) was large enough to swamp out temporal changes observed at the larger community level. If ANOVA tests were significant, means were separated using Fisher's least significant difference (LSD) multiple comparison test. SYSTAT (1997) Version 7.0 was used for all statistical analyses.

Although previous research has suggested that blue vane trap efficiency is not influenced by the nearby availability of floral resources (Stephen and Rao 2007), Pearson correlations were conducted on the number of bees collected per trap with the abundance and richness of blooming forbs in 50 m transect centered on each trap. If trapping efficiency decreased with increased floral resources near traps, the number of bees collected per trap should be significantly and negatively correlated with blooming forb abundance and/or species richness. Correlations were performed for June and July 2008, but not August 2008 because no floral resources were recorded on transects in that month.

## Results

A total of 7124 bees were collected in 2007 and 2034 bees in 2008. For both years combined, 94 species and 117 morphospecies in 27 genera were identified (Appendix 1). 60% of all specimens were identified to species, 37% to morphospecies, and 1% to genus only; 2% were too damaged to identify. The availability of regional generic taxonomic keys varied, resulting in some genera having more morphospecies than others. For example, 99% of specimens of the genus *Bombus* were identified to species and no morphospecies were used. In contrast, *Lasioglossum*, one of the most common genera, had a high proportion of morphospecies, with approximately 36% of all morphospecies belonging to this genus.

### Abundance, richness, evenness, diversity, and sex ratio of the bee community

There were large seasonal differences in the abundance of all bees; adjusted abundance was highest in July of both years, and lowest in August (Table 1). There were also differences between years; adjusted abundance for all bees in June and July decreased from 2007 to 2008 (Table 1).

Species richness showed similar trends as adjusted abundance. The number of species collected for each season ranged from 51 to 183, with the most species collected in July of each year (Table 1). There were also differences between years. For June, more species were collected in 2008 than 2007, but for July, more species were collected in 2007 than 2008. Estimated species richness (Chao 1) displayed the same patterns (Table 1). No inter-annual comparisons could be made for August, since only one year of data was available.

Community evenness remained fairly constant through the seasons and years, ranging from 0.73 to 0.86 (Table 1). Shannon diversity, although highest in July of each year, also did not show large differences through the seasons and years, with values ranging from 3.22 to 3.76 (Table 1).

**Figure 2.**
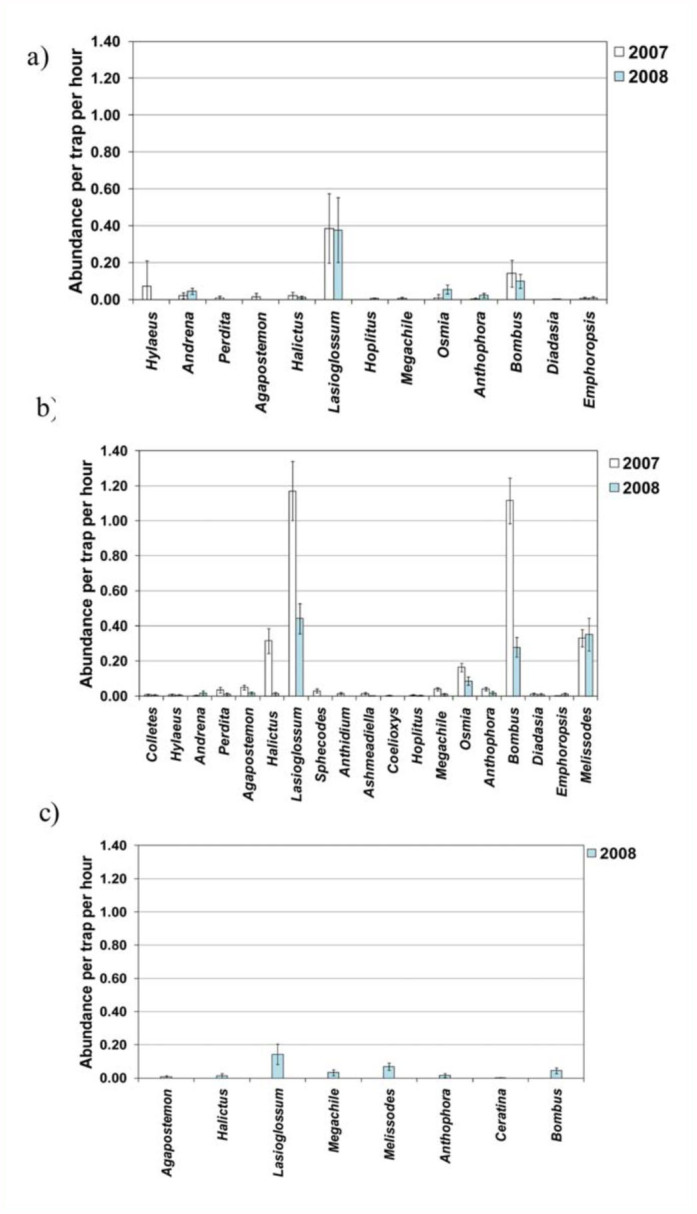
Mean adjusted abundance of common genera in 2007 and 2008 in (a) June, (b) July, and (c) August (2008 only). Error bars represent 95% confidence intervals. High quality figures are available online.

Samples of nearly all genera in all sampling periods were heavily dominated by females (Table 2). The only exceptions were *Sphecodes* in July 2007, when 90% of individuals collected were males, and *Osmia* in June 2008, when 73% were males. Sex ratio also varied seasonally. For example, *Bombus* and *Lasioglossum* started the season with virtually no males, but the proportion of males increased in July of both years. In August 2008, the proportion of males of *Lasioglossum* reached its highest proportion (Table 2). Sex ratio for some genera also varied between years. For example, sex ratios in July for *Bombus* and *Lasioglossum* were more male biased in 2007 than 2008. In contrast, the proportion of male *Melissodes* in July was similar for both years.

### Patterns in bee genera between years

In June, patterns in adjusted abundance (Figure 2a) and relative abundance of genera (Table 3) of both years were similar, with the most common genera being *Lasioglossum* and *Bombus*. However, although the adjusted abundance of *Lasioglossum* in June decreased slightly from 2007 to 2008 (Figure 2a), its relative abundance increased (Table 3). Also, while *Hylaeus* was the third most common genus in 2007, *Andrena* and *Osmia* were the third and fourth most common genera in 2008 (Table 3). In July, the adjusted abundance of the most common genera, except *Melissodes*, decreased from 2007 to 2008 (Figure 2b). General patterns in relative abundance in July were similar in both years, with the most common genera being *Lasioglossum, Bombus*, and *Melissodes* (Table 3). However, *Melissodes* became more dominant and *Bombus* less dominant in 2008, and *Osmia* replaced *Halictus* as the fourth most dominant genus. No comparison of inter-annual patterns could be made for August, since bees were only sampled during that month in 2008.

### Seasonal patterns in bee genera within years

There were several similarities in seasonal patterns in 2007 and 2008. In both years, the adjusted abundance of the most common genera increased from June to July (Figure 2a, 2b) and general patterns in relative abundance were similar, with the most common genus in all seasons of both years being *Lasioglossum* (Table 3). *Bombus* was also common in each month, reaching its peak in relative abundance in July of both years (Table 3). In both years *Melissodes* was uncommon in June and common in July. Although only one year's data are available for August, adjusted abundance of all genera decreased in August (Figure 2b, 2c). Patterns in relative abundance in August 2008 were similar to July of both years, except that *Megachile* occurred at a higher relative abundance in August compared to July (Table 3).

### Total abundance, richness, evenness, diversity, and sex ratio of *Bombus*

Because of the availability of taxonomic keys for *Bombus* (Stephen 1957; Thorp et al. 1983) and because this genus comprises a major component of the Zumwalt bee fauna based on our blue vane trap samples (Table 3), patterns in this genus were examined in greater depth. There were large seasonal differences in the abundance of bumble bees; adjusted abundance was highest in July of both years, and lowest in August (Table 1). There were also differences between years; adjusted abundance for bumble bees in June and July decreased from 2007 to 2008 (Table 1). Bumble bee species richness was the same in June of both years, but decreased in July from 2007 to 2008. 14 species of *Bombus* were identified (Appendix 1), with species richness for each season ranging from 9 to 14 (Table 1). Bumble bee community evenness and Shannon diversity remained fairly constant through the seasons and years, ranging from 0.68 to 0.78 and 1.57 to 1.71, respectively (Table 1).

Sex ratios of bumble bees were examined for July only, since fewer than 30 individuals per species were collected in June or August. In July of both years, almost all *Bombus* species were dominated by workers or queens except for *B. insularis*, which was composed of 79% males in 2007 (Table 4). For the genus overall, there was also a distinct difference between 2007 and 2008 in the proportion of females that were queens in June, with most females being workers in 2007, but most being queens in 2008 (Table 2).

### **Patterns in *Bombus* species between years**

**Figure 3.**
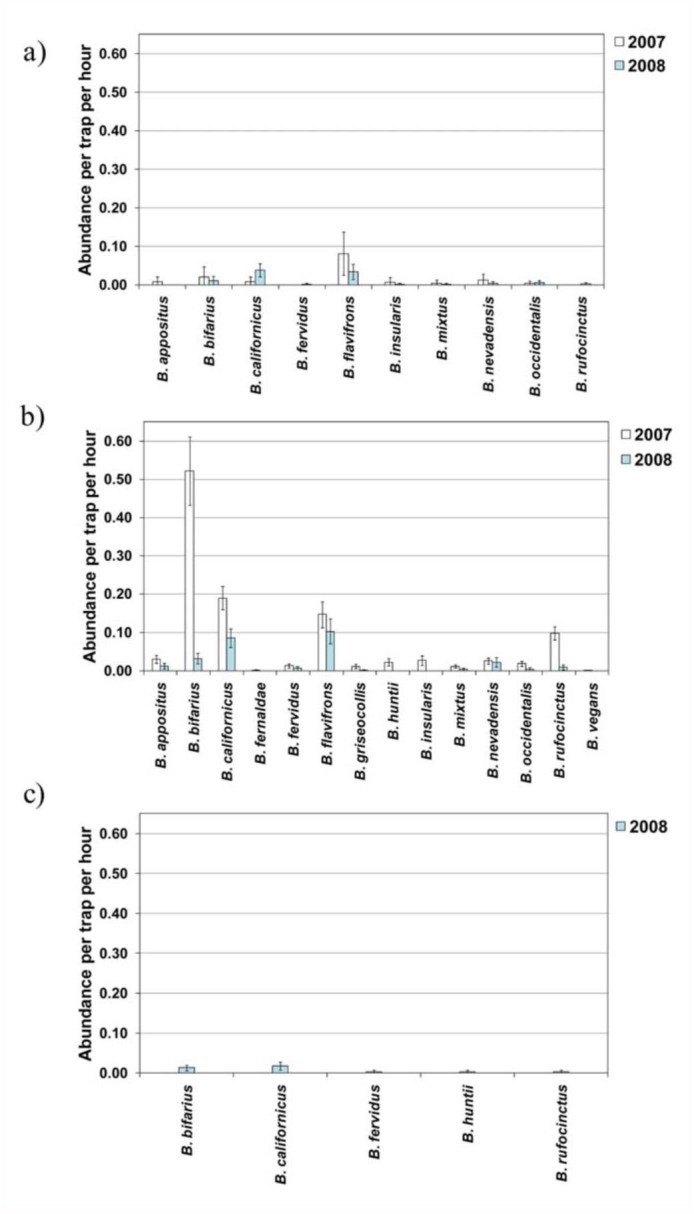
(a) Mean adjusted abundance of common *Bombus* species in 2007 and 2008 in (a) June, (b) July, and (c) August (2008 only). Error bars represent 95% confidence intervals. High quality figures are available online.

Although there were some similarities in patterns of *Bombus* species in 2007 and 2008, there were also strong differences. In June of both years, adjusted abundance was similar and comparatively low for all species (Figure 3a). Patterns in relative abundance in June were also similar, with *B. flavifrons* being the most common species and *B. bifarius* also common in both years (Table 3). However, in 2007, *B. nevadensis* and *B. appositus* were also dominant, but in 2008, *B. californicus* was more common (Table 3). In July, the adjusted abundance of the most common species, except *B. nevadensis*, decreased strongly from 2007 to 2008 (Figure 3b). In addition, the relative abundance of *Bombus* species in July differed between years. Although the same three species, *B. bifarius, B. californicus*, and *B. flavifrons*, dominated in July of both years, their relative abundance differed (Table 3). No comparison of inter-annual patterns could be made for August, since bees were only sampled one year for that month.

### Seasonal patterns in *Bombus* within years

The adjusted abundance of the most common *Bombus* species increased from June to July in both years (Figure 3a, 3b). However, inter-seasonal patterns in relative abundance differed between years. In 2007, the relative abundance of *B. flavifrons* decreased strongly from June to July while the relative abundance of *B. bifarius* increased (Table 3). In contrast, in 2008 the relative abundance of the three most common species was relatively similar from June to July (Table 3). In 2008, adjusted abundance of all *Bombus* species except for *B. huntii* declined from July to August (Figure 3b, 3c), although two species common in July (*B. californicus* and *B. bifarius*) were also common in August (Table 3).

### Spatial and temporal variability in abundance and taxa richness

ANOVA was used to compare temporal variability at the trap level in abundance and estimated species richness of all bees and abundance of bumble bees in this grassland. Time had a significant effect on the total number of bees collected per trap per hour (*F* = 167.4, *df* = 4, 226, *p* < 0.01), the estimated species richness (Chao 1) per trap (*F* = 159.8, *df* = 4**, 226, *p* < 0.01), and the number of bumble bees collected per trap per hour (*F* = 123.7, *df* = 4, 226, p < 0.01). Multiple comparison tests showed that patterns observed at the community level described above were also observed at the trap level (Table 1). Specifically, significantly more bees and bumble bees per trap were collected in July than June of each year (Table 1). Patterns in abundance between years at the trap level were also similar to those observed at the community level. Although adjusted abundances of all bees and bumble bees in June did not differ between 2007 and 2008, both significantly decreased in July from 2007 to 2008 (Table 1). Mean estimated species richness per trap showed the same significant seasonal and yearly patterns as abundance, except that decreases in estimated richness in June from 2007 to 2008 were also statistically significant (Table 1). These results indicate that spatial variability in abundance and richness throughout the study area was not great enough to swamp out temporal patterns evident at the community level.

### Floral resources and weather

**Figure 4.**
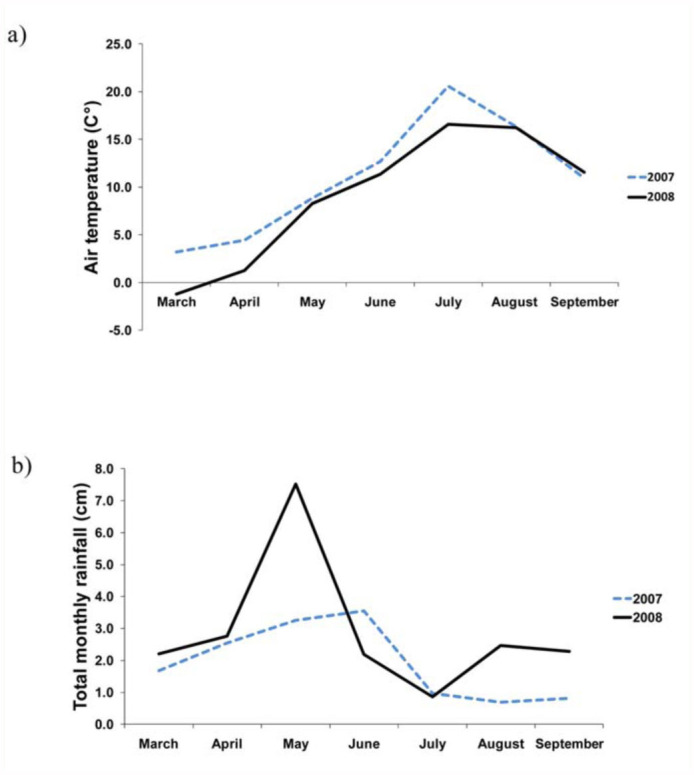
Values of (a) mean average daily temperature (°C) and (b) total rainfall (cm) for spring and summer months in 2007 and 2008. High quality figures are available online.

Both floral resources and weather varied temporally. Blooming forb abundance and richness, which were only examined one year, showed strong seasonal changes. A total of 46 blooming forb species were found on transects adjacent to blue vane traps in 2008, with more stems and species in bloom in June than in July and no forb species blooming on transects in August (Table 5). There was no indication that increases in floral resource availability affected the efficiency of the blue vane traps; neither the abundance nor species richness of blooming forbs adjacent to a trap was associated with significant changes in bees collected in that trap (June 2008, r = 0.19, *p* > 0.05, n = 35 for forb abundance, r = 0.20, *p* > 0.05, n = 35 for forb richness; July 2008, r = -0.03, *p* > 0.05, n = 63 for forb abundance, r = -0.23, *p* > 0.05, n = 63 for forb richness). Average daily temperature peaked in July of both years and was higher in 2007 than 2008 (Figure 4a). In fact, mean temperature was higher in 2007 than 2008 in every month from March to July. Spring 2007 was also drier than 2008 (Figure 4b).

## Discussion

### Native bee communities in the Pacific Northwest Bunchgrass Prairie

Grasslands, in general, are of high conservation value for maintaining bee biodiversity; in temperate systems, grasslands are believed to support a richer bee fauna than other habitats, such as forests (Michener 2007). However, few studies have examined bee communities in North American grasslands, and those that have been conducted have focused on the prairies of the Great Plains (Table 6). Although sampling methods used in the Great Plains varied from study to study and some methods may have under-represented certain groups (e.g., pan traps likely underestimate cavity nesters, Westphal et al. 2008), all indicate that Great Plains grasslands support rich and abundant native bee communities. Likewise, in our study, the first to examine native bee communities in the Pacific Northwest Bunchgrass Prairie, 211 species/morphospecies in 27 genera were found. This level is comparable to the richness of Great Plains grassland bee communities (Table 6), indicating that the unique and threatened Pacific Northwest Bunchgrass Prairie provides important habitat for native bee biodiversity and therefore is of key conservation interest.

In contrast to other studies of bee communities in North American grasslands, the adjusted and relative abundance of all common taxa collected were examined. Describing community composition (i.e., the relative abundance of taxa) is important in understanding and conserving native pollinator communities because richness and diversity indices alone provide a limited picture of native pollinator communities, and may mask or under-represent significant patterns of change, as discussed below and by Williams et al. (2001). In addition, simply reporting absolute abundance of select taxa without information about the total abundance of all bees does not provide information about the relative importance or dominance of those taxa in the overall bee community, and how that dominance may be changing through time.

The bee community was heavily dominated by three genera: *Bombus, Lasioglossum*, and *Melissodes*. The genus *Bombus* consists of bumble bees, which are mid- to large-sized bees (0.9–2.2 cm) that are mostly primitively eusocial and generally abundant in cooler, higher altitude habitats (Michener 2007). Bumble bees are typically generalist foragers and commonly nest in the ground, such as in rodent nests, or in cavities under bunchgrass or other vegetation. Bumble bee colonies are usually annual, and are started in early spring by the queen, who mates in the preceding fall. In contrast, *Lasioglossum*, commonly called sweat bees, is a large genus of primarily small bees (< 0.8 cm) that range from solitary to social. Most are generalist foragers, with a long flight season, and members of this genus typically nest in burrows excavated in banks or flat soil (Potts and Willmer 1997). As with bumble bees, most females mate in fall and then overwinter until good weather in spring, when they emerge to establish their nests (Michener 2007). *Melissodes*, or long-horned bees, are small to medium-sized (0.8–1.8 cm) bees, many of which are specialists on plants in the Asteraceae family. Many are solitary, some are communal, most nest in the ground, and most fly during summer and fall.

The composition of this community is difficult to compare in much detail with other North American grasslands, since little information has been published on the relative abundance of individuals at any taxonomic level in other grassland systems in North America. Kwaiser and Hendrix (2008) do list the relative abundance of the six most common species in their study, which composed 52% of the fauna. Of these, three species of *Lasioglossum* comprised 24% of the fauna, and one species each of *Melissodes, Hylaeus*, and *Augochlorella* comprised 10%, 7%, and 10%, respectively. Thus, *Lasioglossum*, and *Melissodes* and *Hylaeus* to a lesser extent, are dominant taxa in this Iowan native bee community, a pattern also found in our study. Tepedino and Stanton (1981) do not directly report the relative abundance of different genera or species, although they do report absolute abundance of *Bombus* for each year. However, without knowing the total number of bees collected in their study, it is difficult to compare the dominance of *Bombus* between their and our study. Although Kearns and Oliveras (2009) list the three most abundant species (generalists *A. mellifera, Augochlorella striata*, and *Halictus ligatus*), no information on the relative abundance of genera or species is presented. In contrast to their study, no *A. mellifera* was found in our study. Although this may potentially be a bias of blue vane traps (Stephen and Rao 2005, 2007), as discussed below, extensive hand-netting in the Zumwalt Prairie has yet to produce a single specimen of this introduced species (DeBano and Kimoto, unpublished data), suggesting it is uncommon in the area.

### Temporal variability in bee communities

Bee faunas can vary greatly in time (Williams et al. 2001; Michener 2007). The native bee community in the Zumwalt Prairie showed strong seasonal and inter-annual variation. Seasonally, the highest total abundance, richness, and diversity were found in July of both years. In addition, there were large seasonal differences in the dominance of particular genera and bumble bee species. For example, although *Lasioglossum* was dominant throughout the range of collecting dates in both years, other genera, such as *Bombus*, were dominant in early to mid season, while others, such as *Melissodes* and *Megachile*, became more dominant in late season. Many of these patterns are consistent with the general phenology associated with each genus, as discussed above. However, even within a genus, species showed different patterns. For example, species within *Bombus* showed large seasonal fluctuations, with some species, such as *B. flavifrons*, peaking in the early to mid season, and other species, such as *B. bifarius*, becoming more dominant late in the season. Whether these trends also occur in other North American grasslands is unknown, since no information on how relative abundance of community composition changes within the season has been reported (Tepedino and Stanton 1981; Reed 1995; Hines and Hendrix 2005; Davis et al. 2008; Kwaiser and Hendrix 2008; Kearns and Oliveras 2009).

Seasonal variation in bee communities is believed to be primarily the result of changes in floral resource availability and weather (Tepedino and Stanton 1981; Michener 2007; Kearns and Oliveras 2009). However, with regard to floral resource availability, blooming forb abundance and richness were highest in June, not in July when abundance, richness, and diversity of native bees were highest. One possible explanation for the asynchronicity between blooming forb abundance and richness and bee abundance and richness is that bees may not be limited by floral resources in July. Tepedino and Stanton (1981) provided evidence suggesting that bees are often not limited by floral resources in a Great Plains prairie. Weather may also have played a key role; as evidenced by Figure 4, June is often cold and wet. Bees will delay emergence and/or decrease activity in inclement weather, regardless of how many floral resources are available (Michener 2007). Seasonal variation in genera and species composition of native bee communities is also expected, not only because of variation among taxa in their ability to tolerate inclement weather (e.g., bumble bees can fly in colder and windier conditions than other bees (Goulson 2010)), but also because of variation in the degree of specialization and the phenology of plants upon which specialists depend (Michener 2007).

Inter-annual differences in native bee communities on the Zumwalt Prairie were also pronounced. Although adjusted abundance, richness, and diversity in June between years did not differ strongly, taxa composition and proportion of queens and sex ratio of some taxa did. Differences between years were even more pronounced in July. Adjusted abundance, richness, and diversity decreased in 2008, and community composition varied, with *Bombus* becoming less dominant and *Melissodes* more dominant.

As with seasonal variation, weather also appears to play a major role in explaining inter-annual differences in bee fauna in both June and July. June 2008 was colder and wetter, which delayed phenology of flowering forbs on the prairie by two weeks or more (Dingeldein et al. 2010). Bee phenology also appeared to be delayed in 2008, potentially as a direct response to cooler/wetter weather, or an indirect response to delayed plant phenology, or a combination of both. Evidence indicating that differences in bee communities were driven by delayed phenology as an indirect or direct response to weather include the collection of genera, such as *Andrena* and *Osmia*, that are typically active early in the season (Wojcik et al. 2008 ) in June 2008, and their absence in June 2007 (when we presumably first sampled after their peak), and the apparent lag in peaks of *Bombus* species in 2008 compared to 2007. The proportion of *Bombus* queens collected in 2007 and 2008 also suggests delayed phenology. Queens generally engage in most flight activity early in the season until workers are generated and take over foraging duties (Michener 2007). In June 2007, 91% of *Bombus* females were workers, while in June 2008 98% of *Bombus* females were queens.

Although several studies of native bees in North American grasslands have included more than one year's sampling (Tepedino and Stanton 1981; Reed 1995; Hines and Hendrix 2005; Davis et al. 2008; Kwaiser and Hendrix 2008; Kearns and Oliveras 2009), most combine data across years. Only one study describes inter-annual variation in grassland bee communities. Tepedino and Stanton (1981) found large changes in bee communities in Wyoming from one year to another that varied spatially. These changes included a steep decline in bumble bee abundance (but not in richness) during the second year, with only 13% of the *Bombus* collected compared to the previous year. They speculate that these differences were driven by changes in floral resources, which declined in the second year of the study. They hypothesize that bumble bees may have dispersed to live and forage in other areas in response to the decrease in floral abundance at that site, and suggest this response is particularly likely for bumble bees given the high energy needs of these relatively large bees. Decreased floral resources may also explain the decreased abundance of bumble bees on the Zumwalt Prairie in 2008. Because of the delayed flowering of many plants, queens may have shifted their nesting to agricultural areas within 15 km of the preserve, where flowering crops may have provided a more constant floral resource than native plants on the prairie that year. The dispersal capabilities of the bumble bee species found in the Zumwalt Prairie are unknown, and detection and measurement of long distance dispersal in bumble bees is difficult. However, evidence suggests that some bumble bee species are capable of longdistance dispersal flights of up to 30 km (Goulson 2010). Although much attention has been directed at understanding the benefit of native areas next to croplands for enhancing pollinator activity for crops, in some cases, nearby croplands may provide an alternative resource in years when native plants are experiencing delayed and reduced flowering (Rao and Stephen 2010). More research on the potential interaction between agricultural and uncultivated habitats in supporting native bee communities is needed.

An alternative explanation to weather for the decrease in abundance of many genera in 2008 compared to 2007 is that sampling efforts of the previous year depressed populations the following year. This explanation is unlikely for several reasons. First, other taxa that were not destructively sampled showed similar trends. For example, the most common grassland bird species, Savannah Sparrow (*Passerculus* *sandwichensis*), also clearly decreased from 2007 to 2008 (and then increased the following year) (T. Johnson, personal communication). Second, although the total number of bees collected in 2007 was high, the rate of collection per trap was fairly low (maximum = 3.44 bees per hour in July), with each trap operating for two days or less during each sampling period, and a trap density of only one trap per 10 ha.

This study highlights the importance of considering phenology when monitoring bees. Our results support those of others (Williams et al. 2001) that native bee communities are diverse and greatly variable in time and space. In this study, spatial variability was not high enough to swamp out temporal patterns when analyzed at the trap level. Thus, care must be taken when designing long-term monitoring protocols to ensure that sampling occurs during the same point of phenology of bee communities, not simply at the same date year after year. Our study demonstrates that even a few weeks between sampling periods can make a very large difference in abundance, species richness, and community composition, and that phenology can vary substantially from one year to another. Thus, in an ideal situation, initial studies of seasonal variation for monitored sites should be conducted, and attempts to identify cues to initiate sampling identified. These cues may be the presence of early (or late) season taxa, the presence (or absence) of queens outside the nest in eusocial species, or, by proxy, the blooming of plants whose phenologies are closely tied to bee community phenology. Without this information, long-term datasets will be difficult, if not impossible to interpret, as yearly increases or decreases may simply reflect seasonal variation in bee phenology.

### Availability of pollinators for a threatened grassland plant species

The Zumwalt Prairie has the largest populations of the threatened plant, Spalding's catchfly *(S. spaldingii)* (United States Fish and Wildlife Service 2007) and evidence indicates that bumble bees are key pollinators for this species, both at the Zumwalt Prairie (Tubbesing et al. in review) and at other locations in the region (Lesica and Heidel 1996). Our results show that bumble bees are abundant on the prairie, particularly in July, the month of peak flowering for *S. spaldingii* (Taylor et al. 2012). The Zumwalt Prairie may support an abundant bumble bee fauna because of its large size and its relatively intact status. Preserving large, relatively intact grasslands may not only be important for preserving native bee diversity, but may also benefit the native plants that depend on them.

### Sampling native bee fauna in grasslands with blue vane traps

This study also demonstrated the potential usefulness of the blue vane trapping method for sampling native bees in grassland habitats. An essential step in conserving native bee fauna is identifying which species are declining in a given area. Thus, the importance of monitoring bee communities through time has long been recognized and the need for repeatable, standardized sampling methods that allow comparisons of trends across time and space is well appreciated (e.g., São Paulo Declaration on Pollinators 1999; LeBuhn et al. 2003; Westphal et al. 2008). Each technique used to sample native bees has advantages and disadvantages, including logistical issues and certain types of biases inherent in all sampling techniques (Westphal et al. 2008; Droege et al. 2010), and the magnitude of these will vary with many factors, including habitat type, personnel involved in the project, and the bee fauna being sampled. Sampling native bees in North American grasslands can exacerbate some of the disadvantages of traditional sampling methods. Although these grasslands may appear superficially homogenous, many show a large degree of heterogeneity with regard to both soils and vegetation. This presents a challenge in efficiently and adequately sampling a large, variable landscape with most traditional techniques, such as hand-netting and pan-trapping, which are logistically better suited for smaller spatial scales.

Blue vane trapping overcame many of the logistical problems associated with working in these large variable landscapes. Relatively few traps were needed to collect large numbers of bees. The traps are easy to work with in field conditions, being light weight and not requiring liquids to be carried, or fluid levels to be monitored for evaporation. They do not result in wet specimens; in fact, specimen condition was excellent. The traps are selective (more than 80% of all specimens collected in our study were bees), which reduces the amount of time needed to sort through samples with non-focal individuals. In addition, unlike many active collecting methods, the effectiveness of the method is not affected by the experience and capabilities of the sampler (Westphal et al. 2008). Finally, our data also suggests that the proximity of floral resources to the trap does not decrease the efficiency of the trap, a result consistent with other work (Stephen and Rao 2007). All of these characteristics make the technique very applicable for use by non-profit and volunteer organizations world-wide. Finally, blue vane traps do not necessarily kill specimens, allowing for the potential of catch and release. This may reduce the impact of trapping on bees, especially for long term studies (Stephen and Rao 2005), an option that may be particularly beneficial for conservation-related research. However, more research needs to be conducted on the viability of capture and release of pollinators collected with blue vane traps, including quantifying the amount of time required for handling, investigating mortality rates under varying environmental conditions, and determining the best methods to prevent the recapture or double-counting of released individuals.

With regard to trap biases against certain taxa, previous work suggests that blue vane traps are more effective, both in terms of number of individuals and the number of species collected, than net sampling or vacuuming; only *A. mellifera* was clearly underrepresented by blue vane trapping (Stephen and Rao 2007). Nevertheless, like all sampling methods, blue vane traps potentially over- or under-represent individuals of particular species or sex. Thus, we cannot say that blue vane traps give an unbiased assessment of the native bee community. However, as pointed out by Droege et al. (2010), this is a problem common to all native bee sampling techniques, and no studies that we are aware of have yet demonstrated an independent and unbiased method of describing bee communities with which to compare different sampling methods. In addition, as long as biases of a particular method are consistent through time, the method will be useful for monitoring the relative changes in bee communities it samples through time. Nevertheless, further research comparing this and other sampling methods is needed to determine which method or combination of methods is most effective at characterizing native bee communities in North American grasslands.

### Conclusions and broader implications for conservation

This study showed that the Pacific Northwest Bunchgrass Prairie, like other grasslands in North America, supports a rich and diverse native bee fauna, including species, such as the western bumble bee, *B. occidentalis*, that have virtually disappeared from other parts of its range (Rao and Stephen 2007; Rao et al. 2011). Like grasslands world-wide, the remnants of Pacific Northwest Bunchgrass Prairie continue to be threatened by various types of human activities including conversion to cropland, improper livestock management, and invasions by non-native plants (Samson and Knopf 1994; Brennan and Kuvlesky 2005). Both conserving remaining intact habitats and restoring disturbed portions of this type of prairie will be important for conserving native bees and the plants, such as Spalding's catchfly, that depend on them.

This study also illustrates the importance of timing monitoring efforts aimed at determining trends in pollinator communities. Sampling for long-term monitoring should occur at the same point in bee community phenology. Sampling efforts could potentially be tied to the phenology of common early, middle, or late season bee species, to queen/worker ratios in social species, or to the phenology of floral resources or some closely related surrogate. In addition, this study highlights the importance of reporting species composition of communities (i.e., some measure of abundance of each taxa), and how they vary through seasons and years, to better understand trends in bee communities through time and space. Focusing only on richness and total abundance of all bees or select groups can mask important changes in bee communities. Finally, this study demonstrates the potential usefulness of a promising sampling technique for native bees that has never been used in North American grasslands: the blue vane trap. This method appears to be well-suited for sampling native bees in these grasslands because it is economical, does not require lethal sampling, and can be easily implemented in large expanses.

**Table 1.**
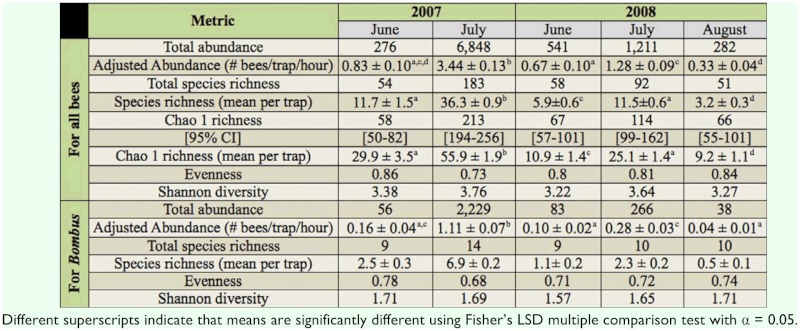
Abundance, richness, evenness, and diversity of all bees and of bumble bees found in the Zumwalt Prairie.

**Table 2.**
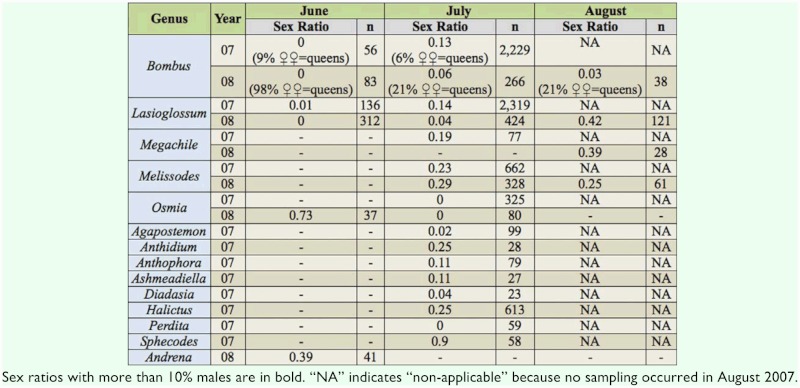
Sex ratios, as expressed by proportion of males, of common genera (> 20 specimens) in each season.

**Table 3.**
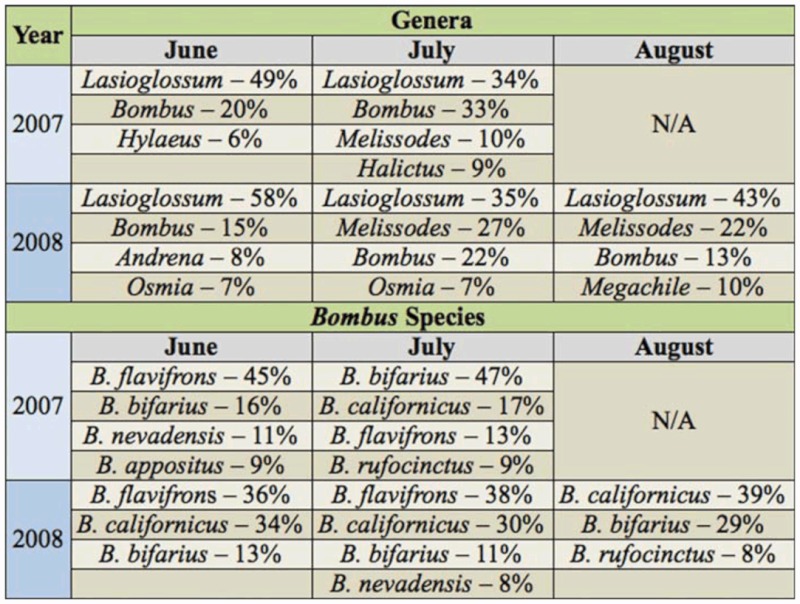
Relative abundance of most common genera and *Bombus* species (> 5%) in each season of each year.

**Table 4.**
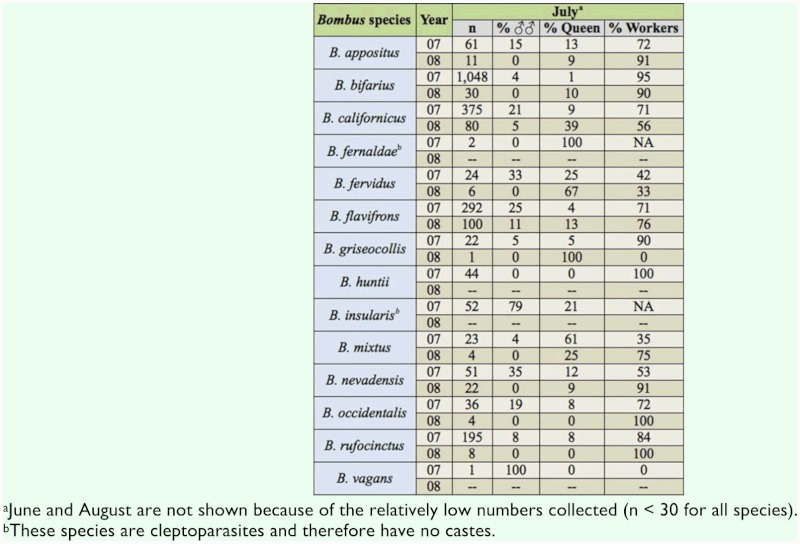
Percentage of males, queens, and workers in *Bombus* species in July 2007 and 2008.

**Table 5.**
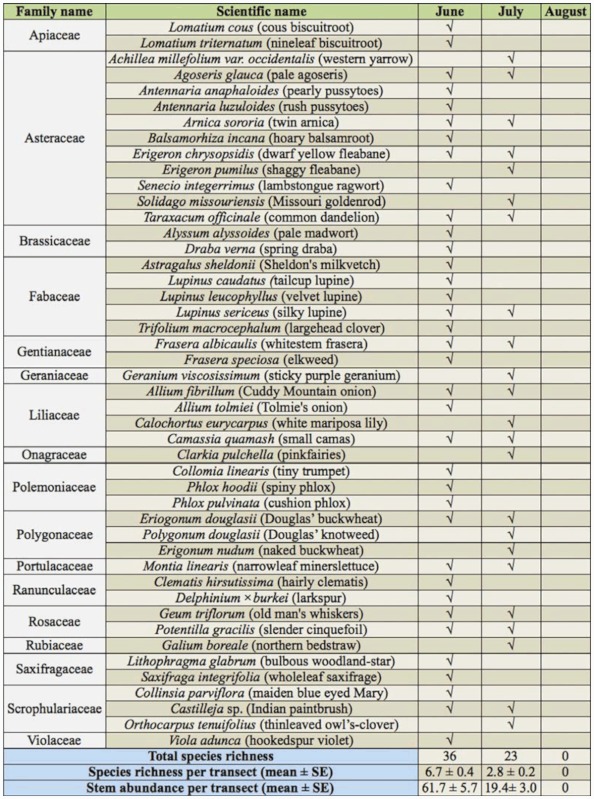
List of plant species blooming within 50 m of blue vane traps during each sampling period in 2008.

**Table 6.**
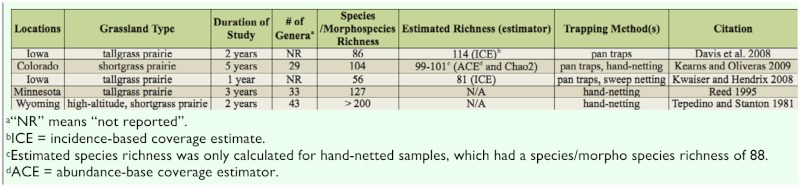
Summary of studies of bee communities in North American grasslands. All studies were conducted in the Great Plains region of the U.S.A.

## Figures and Tables

**Appendix 1.**
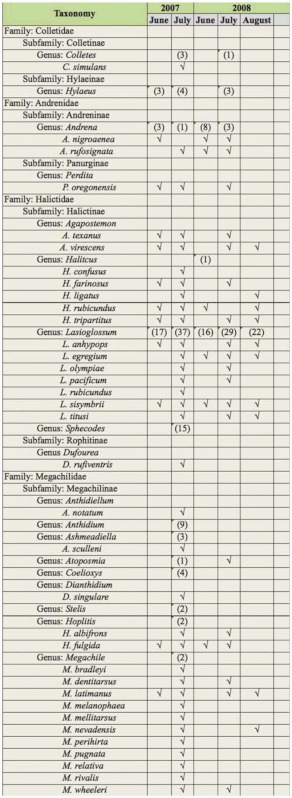
List of bee taxa found in the Zumwalt Prairie and their presence/absence for each season.

## Abbreviation

ANOVA,: analysis of variance;
LSD,: least significant difference
